# Machine Learning for Predicting Critical Events Among Hospitalized Children

**DOI:** 10.1001/jamanetworkopen.2025.13149

**Published:** 2025-05-30

**Authors:** Sierra Strutz, Huan Liang, Kyle Carey, Fereshteh Bashiri, Priti Jani, Emily Gilbert, Julie L. Fitzgerald, Nicholas Kuehnel, Maya Dewan, L. Nelson Sanchez-Pinto, Dana Edelson, Majid Afshar, Matthew Churpek, Anoop Mayampurath

**Affiliations:** 1 Department of Biostatistics & Medical Informatics, University of Wisconsin-Madison, Madison; 2 Department of Medicine, University of Chicago, Chicago, Illinois; 3 Department of Medicine, University of Wisconsin-Madison, Madison; 4 Department of Pediatrics, University of Chicago, Chicago, Illinois; 5 Department of Medicine, Loyola University Medical Center, Chicago, Illinois; 6 Department of Pediatrics, Loyola University Medical Center, Chicago, Illinois; 7 Department of Emergency Medicine, University of Wisconsin-Madison, Madison; 8 Division of Critical Care Medicine, Department of Pediatrics, University of Cincinnati, Cincinnati, Ohio; 9 Department of Pediatrics, Ann & Robert H Lurie Children’s Hospital of Chicago, Chicago, Illinois

## Abstract

**Importance:**

Unrecognized deterioration among hospitalized children is associated with a high risk of mortality and morbidity. The current approach to pediatric risk stratification is fragmented, as each hospital unit (emergency, ward, or intensive care) uses different tools for predicting specific outcomes.

**Objective:**

To develop a machine learning model for the early detection of deterioration across all units, thereby enabling a unified risk assessment throughout the patient’s hospital stay.

**Design, Setting, and Participants:**

This retrospective cohort study used data from pediatric (age <18 years) admissions to inpatient and intensive care units at 3 tertiary care academic hospitals. Data were analyzed from January 2024 to March 2025.

**Main Outcomes and Measures:**

The primary outcome was critical events, defined as invasive mechanical ventilation, administration of vasoactive medications, or death within 12 hours of an observation.

**Results:**

The cohort included 135 621 patients (mean [SD] age, 7 [6] years; 60 376 [44.5%] female). Patient age, hospital unit, vital signs, laboratory results, and prior comorbidities were used to derive a regression-based model, an extreme gradient-boosted machine (XGB) model, and 2 deep learning models. Data from 2 hospitals were used as a derivation cohort, while patients in the third hospital constituted the hold-out external test cohort. The XGB model was the best-performing machine learning model, outperforming 2 existing ward-focused models in terms of discrimination (*C* statistic: XGB, 0.86; ward-focused models, 0.82 [*P* < .001] and 0.70 [*P* < .001]) and the number needed to alert (at an example 80% sensitivity: XGB, 6 ward-focused models: 9 and 11). The deep learning models did not exhibit improved performance. The XGB model performed better or equivalent to models trained for a specific hospital unit.

**Conclusions and Relevance:**

This retrospective cohort study describes the development of a novel hospitalwide model for continuously predicting the risk of critical events through the entirety of a child’s stay. The model facilitated a unified framework for risk assessment in a pediatric hospital.

## Key Points

**Question:**

Can a hospitalwide machine learning model accurately predict critical events among hospitalized children across emergency, ward, and intensive care units?

**Findings:**

In this cohort study including data from 135 621 pediatric patients, a gradient-boosted machine learning model demonstrated superior performance in predicting hospitalwide critical events, defined as mechanical ventilation, administration of vasoactive drugs, or mortality, compared with clinical standards and other machine learning models. The gradient-boosted machine learning model also showed equivalent or better performance than models trained for a specific hospital unit.

**Meaning:**

These findings suggest that a gradient-boosted machine learning model can continuously assess risk for children as they progress through their hospital stay, potentially improving outcomes for children.

## Teaser

This cohort study describes the development and testing of the pediatric Critical Event Risk Evaluation and Scoring Tool, a machine learning model for the early detection of deterioration among pediatric patients across all hospital units.

## Introduction

Physiological decompensation in hospitalized children that requires the initiation of mechanical ventilation or administration of vasoactive medications increases the risk for mortality.^1,2,3,4,5,6^ Survivors of these events remain at risk for long-term functional or neurodevelopmental impairment extending months after hospital discharge.^7,8,9,10,11,12^ As many of these events are unrecognized,^13^ early identification of at-risk children and timely intervention are necessary to improve outcomes.^14,15^

Risk prediction models have been developed to detect early signs of deterioration in hospitalized children and serve as a foundation for clinical decision support (CDS) tools. However, risk stratification in a pediatric hospital is splintered across unit-specific silos, each focused on distinct outcomes.^16^ For example, tools in the pediatric emergency department (ED) primarily predict triage or conditions, such as sepsis.^17^ In contrast, studies in the pediatric intensive care unit (ICU) target estimating the risk of mortality,^18^ organ dysfunction,^19,20^ and resuscitation events.^21^ On the pediatric ward, tools, such as the Bedside Pediatric Early Warning System (Bedside PEWS),^22,23^ our machine learning–based pediatric Calculated Assessment of Risk and Triage (pCART) tool,^24^ and other scores,^25,26^ have been developed to predict the risk of deterioration. The availability of these disparate models across hospital units causes clinicians to be presented with different risk scores targeted toward various outcomes, which shift with a patient’s location within the hospital.^16^ Moreover, implementing these specialized models outside their intended hospital unit may diminish their utility.^27,28,29^ Overall, pediatric hospitals use a compartmentalized approach to risk stratification that could lead to fragmented care, hindering a cohesive assessment of deterioration through a child’s hospital stay.

The objective of this study is to develop a new model for the prediction of a critical event, defined as invasive mechanical ventilation, vasoactive medication administration, or in-hospital death, within 12 hours of any vital sign or laboratory result during a child’s hospitalization. We hypothesize that a machine learning model trained on multicenter electronic health record (EHR) data will accurately predict critical events in children during external validation.

## Methods

This cohort study was approved by institutional review boards at each participating site with a waiver of informed consent because it was determined to be of minimal risk. We followed the Transparent Reporting of a Multivariable Prediction Model for Individual Prognosis or Diagnosis plus Artificial Intelligence (TRIPOD+AI) reporting guidelines for reporting the development and evaluation of our models.^30^

### Study Population

We conducted a retrospective study of all pediatric (age <18 years) admissions to the ward and the ICU across 3 tertiary care centers: University of Chicago Comer Children’s Hospital (hereafter, *UC*; 2009-2019), Loyola University Ronald McDonald Children’s Hospital (hereafter, *Loyola*; 2006-2020), and the American Family Children’s Hospital at University of Wisconsin-Madison (hereafter, *UW-Madison*; 2009-2021). Birth encounters, including neonatal ICU admissions, were excluded. Data for this study were extracted from each hospital’s EHR (EPIC Systems) database.

### Outcome and Predictors

The primary outcome of interest was a critical event, defined as a binary composite outcome of invasive mechanical ventilation, vasoactive drug administration, or in-hospital mortality, within 12 hours of a vital sign or laboratory result observation in the ward, ED, or ICU.^1,2,31^ The primary outcome was censored if the patient experienced mechanical ventilation or was administered vasoactive medications within the prior 24 hours. Outcomes in the operating room were not considered in this study. However, the censoring logic was still implemented, in that all outcomes in the 24-hour period of a mechanical ventilation or vasoactive medication event in the operating room were censored.

Only ward, ED, or ICU observations were used for model derivation and validation. Model variables included age, location of the patient (ED, ward, or ICU), vital signs, the fraction of inspired oxygen (Fio_2_), neurological assessment measure using the alert-verbal-pain-unresponsive scale, count of prior comorbidities (categorized as 0, 1, or >1), and results from standard laboratory tests. The complete variable list, distribution, and percentage of admissions with missing values across all sites are provided in eTable 1 in Supplement 1. These measures were chosen for their ubiquity across units, routine capture, and prior evidence.^24^ Comorbidities were calculated using the pediatric complex chronic condition criteria.^32,33^ The delivery method of Fio_2_ was not included as a variable due to inherent inconsistency in coding across sites and the added complexity of practice changes that cause the delivery method and documentation to vary over time. Descriptive statistics (*t* tests for age, Wilcoxon rank-sum tests for length of stay, and χ^2^ for categorical variables) were used to compare patients who experienced the primary outcome with those who did not at each site.

### Derivation of Prediction Models

Data were split by geographic location into derivation cohort (UC and Loyola) and a hold-out external test cohort (UW-Madison). We further split the derivation data longitudinally into temporal derivation (2017 and earlier) and temporal validation (after 2017). Similar to prior work,^24^ we used a discrete-time survival analysis framework to create a regularized logistic regression (LR) model and an extreme gradient-boosted machine (XGB) model.^34,35,36^ Our approach is illustrated in eFigure 1 in Supplement 1 and explained in the eMethods and eTable 2 in Supplement 1. The window of 12 hours for predicting critical events was chosen to maintain consistency with prior work.^24,37^ The temporal validation and the external test cohorts were not blocked when evaluating model performances. The LR model was regularized using ridge and least absolute shrinkage and selection operator regularization approaches.

We also created 2 recurrent neural network (RNN) models to explore if deep learning models perform well in predicting critical events 12 hours in advance. First, we created an RNN that used the same variables as our non–deep learning models as input, thereby testing the possibility that using longitudinal information within individual variables would improve performance. Second, we created a separate RNN that used the output of the hyperparameter-optimized XGB model and the difference from the most recent time measurement as input (referred to as the XGB-RNN tandem model). This allowed us to test whether the trend of a risk score could be used to improve the performance of predicting critical events compared with the risk score (eMethods in Supplement 1).

Details regarding hyperparameter optimization and addressing of missing values are provided in the eMethods in Supplement 1. As baseline models, we used a modified version of Bedside PEWS (ie, only using vital sign measurements) and pCART.

### Statistical Analysis

Final models were used to calculate predicted probabilities in the temporal validation and external test datasets. Model-predicted probabilities were generated for every new observation of a vital sign reading or a laboratory result. The primary metric for assessing model performance was discrimination, evaluated using the area under the receiver operating characteristic curve (AUC) on the temporal validation and external test cohorts. Model AUCs (including 95% CIs) were compared using the nonparametric DeLong method.^38^ Additionally, we evaluated the area under the precision-recall curve of all models in the temporal validation and external test data. We then analyzed the efficiency curve of the best-performing model within the external test data by plotting the percentage of observations crossing a chosen alert threshold across different sensitivity values within the ED, ward, and ICU. We also compared the sensitivity and specificity for the best-performing model and pCART at various model thresholds on the external test cohort. Within the external test cohort, we also compared the number needed to alert (NNA) to detect a true positive at different sensitivities for the XGB model, pCART, and Bedside PEWS, calculated using maximum values per hospital admission, similar to prior work.^24^ The NNA is calculated as the inverse of the positive predictive values. At higher sensitivities, the total number of alerts will increase. However, the rate of false positives will also increase, causing a reduction in positive predictive value and a subsequent increase in the NNA. We estimated the overall variable importance for the best-performing model using information gain. Importance was normalized to the most important variable. Variable importance was also assessed in a single-patient instance using Shapley values, which measure the contribution of each variable for the prediction for a single observation, calculated from the DALEX package.^39,40^

To determine whether the best-performing hospitalwide model had better performance in predicting critical events compared with models developed using unit-specific data, we developed 3 unit-specific XGB models (eMethods in Supplement 1). Finally, we conducted a sensitivity analysis of the best-performing hospitalwide model within patient subgroups based on year of admission (before vs during the COVID-19 pandemic), patient age, and number of prior comorbidities. Analyses were conducted from January 2024 to March 2025. All analyses were performed using R software version 4.4.0 (R Project for Statistical Computing) and Python version 3.9.18 (keras 3.7.0, tensorflow 2.18.0). A 2-sided *P* < .05 threshold was used to assess significance.

## Results

### Study Population

Our cohort included 135 621 patient admissions (mean [SD] age, 7 [6] years; 60 376 [44.5%] female) across all 3 sites. Among 55 059 patients in the UC cohort, 2541 patients (4.6%) experienced at least 1 critical event during their hospital stay. Of 38 913 patients in the Loyola cohort, 779 patients (2.0%) experienced at least 1 critical event during their hospital stay. The UW-Madison cohort, ie, our external test dataset, included 41 649 patients, of whom 2543 patients (6.1%) experienced at least 1 critical event. The derivation dataset had a total of 701 817 twelve-hour blocks, of which 3449 (0.5%) had a positive primary outcome, 615 391 (87.7%) had a negative primary outcome, and 82 977 (11.8%) were censored. Table 1 compares the patient characteristics and hospital outcomes for all patients with and without the primary outcome.

**Table 1.** Site-Specific Comparisons of Characteristics and Outcomes Observed Between Patient Admissions Who Did and Did Not Experience at Least One Critical Event During Their Hospital Stay

Characteristic or outcome	Patients, No. (%)					
	UC		Loyola		UW-Madison	
	With critical events (n = 2541)	Without critical events (n = 52 518)	With critical events (n = 779)	Without critical events (n = 38 134)	With critical events (n = 2543)	Without critical events (n = 39 106)
Age, mean (SD), y	6 (6)^a^	7 (6)	7 (6)^a^	6 (6)	5 (6)^a^	7 (6)
Sex						
Male	1454 (57.2)	28 863 (55.0)	463 (59.4)	21 872 (57.4)	1336 (52.5)	21 257 (54.4)
Female	1087 (42.8)	23 655 (45.0)	316 (40.6)	16 262 (42.6)	1207 (47.5)	17 849 (45.6)
Race						
Black	1565 (61.6)^a^	31 240 (59.5)	222 (28.5)	9245 (24.2)	289 (11.4)	3777 (9.7)
White	569 (22.4)	14 350 (27.3)	371 (47.6)	17 916 (47.0)	2057 (80.9)	32 590 (83.3)
Other^b^	407 (16.0)	6928 (13.2)	186 (23.9)	10 973 (28.8)	197 (7.7)	2739 (7.0)
Hispanic	326 (12.8)	6150 (11.7)	294 (37.7)	15136 (39.7)	250 (9.8)	3564 (9.1)
Prior comorbidity count						
0	1475 (58.0)^a^	39 693 (75.6)	466 (59.8)^a^	31 049 (81.4)	707 (27.8)^a^	20 143 (51.5)
1	230 (9.1)	5148 (9.8)	66 (8.5)	3514 (9.2)	465 (18.3)	5802 (14.8)
>1	836 (32.9)	7677 (14.6)	247 (31.7)	3571 (9.4)	1371 (53.9)	13 161 (33.7)
Length of stay, median (IQR), d	8 (3-19)^a^	2 (1-4)	7 (1-19)^a^	2 (1-3)	5 (1-14)^a^	2 (1-4)
Initial hospital location						
ICU	926 (36.4)^a^	4722 (9)	259 (33.2)^a^	4797 (12.6)	689 (27.1)^a^	2850 (7.3)
ED	1238 (48.7)	26 706 (50.9)	355 (45.6)	16 862 (44.2)	1145 (45.0)	15 503 (39.6)
Ward	223 (8.8)	13 780 (26.2)	138 (17.7)	14 544 (38.1)	436 (17.1)	13 538 (34.6)
Other^c^	154 (6.1)	7310 (13.9)	27 (3.5)	1931 (5.1)	273 (10.7)	7215 (18.4)
Mortality	76 (3.0)	NA	22 (2.8)	NA	41 (1.6)	NA
Mechanical ventilation	2311 (90.9)	NA	585 (75.1)	NA	1969 (77.4)	NA
Vasopressors	574 (22.6)	NA	251 (32.2)	NA	891 (35)	NA

Abbreviations: ED, emergency department; ICU, intensive care unit; Loyola, Loyola University Ronald McDonald Children’s Hospital; NA, not applicable; UC, University of Chicago Comer Children’s Hospital; UW-Madison, American Family Children’s Hospital at University of Wisconsin-Madison.

^a^ *P* < .001 compared with patients who did not experience critical events at that site.

^b^ Includes American Indian or Alaska Native, Asian or Mideast Indian, declined/unknown, multiracial, Pacific Islander or Hawaiian Native, and other.

^c^ Includes the operating room, interventional and diagnostic areas (eg, sedation, radiation therapy, radiology, etc.), and other indeterminate hospital locations.

### Model Performances

Table 2 reports model performances on our temporal validation and external test datasets. All hospitalwide models outperformed the modified Bedside PEWS score and our previously developed pCART model in predicting the outcome. The XGB model outperformed the LR model (temporal validation: AUC, 0.89 [95% CI, 0.89-0.89] vs 0.83 [95% CI, 0.83-0.83]; *P* < .001; external test: AUC, 0.86 [95% CI, 0.86-0.87] vs 0.84 [95% CI, 0.84-0.84]; *P* < .001). The XGB model also outperformed the 2 ward-focused models in the internal (Bedside PEWS: AUC, 0.67 [95% CI, 0.67-0.67]; *P* < .001; pCART: AUC, 0.79 [95% CI, 0.78-0.79]; *P* < .001) and external (Bedside PEWS: AUC, 0.70 [95% CI, 0.70-0.70]; *P* < .001; pCART: AUC, 0.82 [95% CI, 0.81-0.82]; *P* < .001) test cohorts. Henceforth, we will refer to this model as the pediatric Critical Event Risk Evaluation and Scoring Tool (pCREST). The deep learning models (the RNN and tandem XGB-RNN) did not have improved AUC performance over pCREST in either evaluation cohort (Table 2). The pCREST model outperformed all models in terms of area under the precision-recall curve in both cohorts (eTable 3 in Supplement 1).

**Table 2.** Performance of Hospitalwide Models at Predicting Critical Events Within the Next 12 Hours Using the Temporal Validation and the External Test Datasets

Model	AUC (95% CI)	
	Temporal validation	External test
Bedside PEWS	0.67 (0.67-0.67)	0.70 (0.70-0.70)
pCART	0.79 (0.78-0.79)^a^	0.82 (0.81-0.82)^a^
Logistic regression	0.83 (0.83-0.83)^a^^,^^b^	0.84 (0.84-0.84)^a^^,^^b^
XGB (pCREST)	0.89 (0.89-0.89)^a^^,^^b^	0.86 (0.86-0.87)^a^^,^^b^
RNN	0.87 (0.87-0.87)^a^^,^^b^	0.84 (0.84-0.85)^a^^,^^b^
XGB-RNN	0.88 (0.88-0.88)^a^^,^^b^	0.85 (0.85-0.86)^a^^,^^b^

Abbreviations: AUC, area under the receiver operating characterstic curve; Bedside PEWS, Bedside Pediatric Early Warning System; pCART, pediatric Calculated Assessment of Risk and Triage; pCREST, pediatric Critical Event Risk Evaluation and Scoring Tool; RNN, recurrent neural network; XGB, extreme gradient-boosted.

^a^ *P* < .001 compared with the Bedside PEWS model.

^b^ *P* < .001 compared with the pCART model.

**Figure 1.**
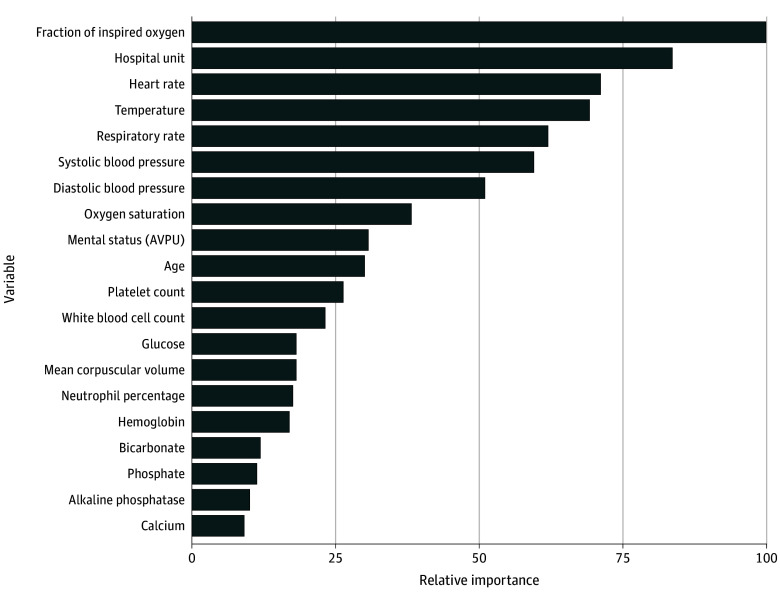
The Top 20 Most Important Variables, as Assessed Using Information Gain, From the pCREST Model for Predicting Critical Events Mental status was measured using the alert-verbal-pain-unresponsive (AVPU) scale.

Figure 1 illustrates the top 20 most important variables for pCREST ranked in descending order of importance. The most important features included the Fio_2_, hospital unit location, heart rate, temperature, and respiratory rate. Platelet count, white blood cell count, and glucose measurements demonstrated high importance among laboratory values.

**Figure 2.**
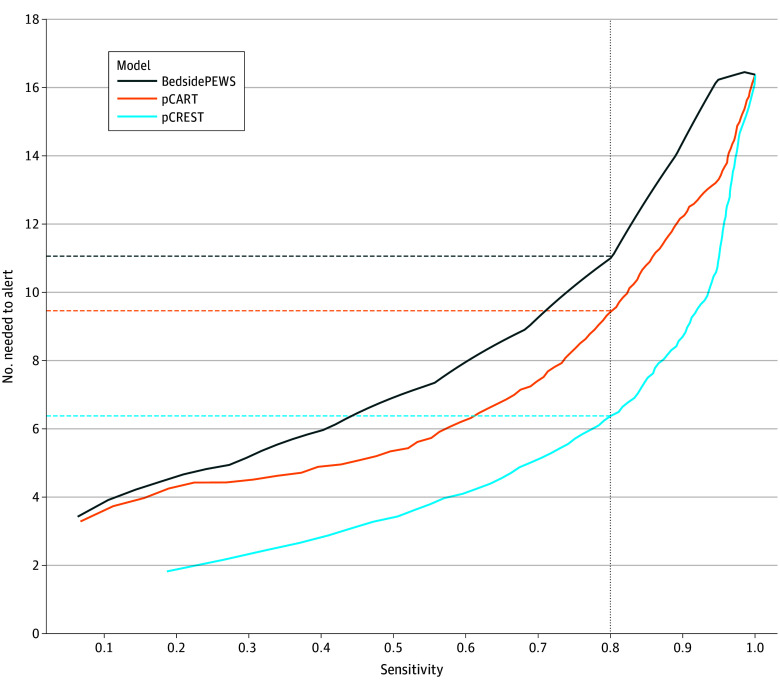
The Number Needed to Alert for Various Sensitivity Values for the Pediatric Critical Event Risk Evaluation and Scoring Tool (pCREST), Pediatric Calculated Assessment of Risk and Triage (pCART), and Bedside Pediatric Early Warning System (Bedside PEWS) in the External Test Cohort at University of Wisconsin-Madison

eFigure 2 in Supplement 1 illustrates variable importance, as indicated by Shapley values, for pCREST scores (indicated as percentile risk between 0 and 100) across several time points for a hospital stay for a patient aged 16 years as they were triaged in the ED, admitted to the ward, and subsequently moved to the ICU before experiencing a critical event. eFigure 3 in Supplement 1 indicates that efficiency curves for pCREST were similar across all units. The sensitivity and specificity measures for pCREST and pCART at various score thresholds are shown in eTable 4 in Supplement 1. The NNA at different sensitivities for pCREST, pCART, and Bedside PEWS are shown in Figure 2. Across most threshold-specific sensitivity measures, pCREST resulted in lower NNA compared with pCART and Bedside PEWS across the hospital. For example, at an example sensitivity of 80%, pCREST would have resulted in 3 and 5 fewer patient-level alerts than pCART (6 vs 9) and Bedside PEWS (6 vs 11), respectively.

eTable 5 in Supplement 1 compares the performance of pCREST and 3 XGB models trained using hospital unit–specific observations within the derivation data on corresponding unit-specific observations in the external test data. As shown, pCREST performance was equivalent to the unit-specific model within the ED and ICU but performed better than the unit-specific models in the ward. The results of our subgroup analysis are provided and explained in eTables 6 to 8 in Supplement 1.

## Discussion

In this cohort study, we developed a new machine learning model, pCREST, to predict the likelihood that a child will experience a critical event within 12 hours of any vital sign or laboratory result observation during hospitalization. Derived using multicenter data, pCREST outperformed other models in terms of discrimination and other clinically relevant metrics during temporal and external validation. Notably, pCREST matched or exceeded the performance of machine learning models trained to a specific unit. The availability of a single hospitalwide risk stratification model allows continuous risk monitoring throughout a patient’s stay, enabling early recognition and timely rescue of children at risk for critical events and streamlined care delivery.

Several studies have proposed risk prediction models that can be integrated into CDS tools within EHRs to identify children in the early phases of deterioration and enable timely interventions. For example, machine learning models in the ED primarily focus on aiding with decisions related to triage.^41,42,43^ Risk stratification models in the pediatric ICU focus on predicting mortality or cardiac arrests,^44,45,46,47^ and risk scores incorporated into CDS tools have been associated with decreased incidences of cardiopulmonary resuscitation events.^21^ Similarly, ward-based models primarily predict ICU transfer by identifying indications of deterioration,^24,26^ and subsequent implementation for real-time risk stratification in the ward was associated with positive patient outcomes.^48^ Despite these advances, model performances have been noted to decline when validated outside their intended hospital unit.^29^ For example, we observed in this study that pCART did not perform well in predicting critical events in patients outside the ward. Given the lack of evidence of generalizability, the current paradigm for early recognition of deterioration is structured to apply to specific outcomes and hospital units, placing patients at risk for fragmented care, which has been associated with adverse outcomes.^49,50^ In addition, the disjointed risk assessment hampers decision-making within the health system, whose leaders do not have adequate information to guide the allocation of resources.^16^ Transitioning to a cohesive, hospitalwide model, such as pCREST, could enhance care continuity, potentially improving outcomes for children and increasing efficiency in hospital workflows.

The pCREST model outperformed clinical standards and machine learning models in predicting hospitalwide critical events several hours in advance. Extension to more advanced deep learning architectures did not improve model discrimination in both the temporal validation and external test cohorts. These findings suggest that incorporating longitudinal trends in vital signs and laboratory data does not provide additional predictive value beyond using the most recent observations for identifying hospitalized children at risk for critical events. We also found that the performance of pCREST was similar to ED- and ICU-specific machine learning models and superior to ward-specific models. This result highlights the ability of pCREST to generalize to patients across units and departments that differ in event rates, clinical care, and workflows.

The numerical output of pCREST indicates the likelihood of the patient experiencing a critical event within the next 12 hours, and the output is generated with every new vital sign or laboratory result recorded in the EHR. The variables used by pCREST are age, standard physiological measurements that are routinely collected, location of the patient within the hospital, and comorbidities from prior encounters, all of which are easily extractable from the EHR and do not impede prospective implementation. The operationalization of pCREST also does not depend on imputation, as it uses the most recent observations for prediction. To enhance interpretation, pCREST outputs can be scaled to a 0 to 100 score, similar to our illustration in eFigure 2 in Supplement 1 or our implementation of pCART,^48,51^ and incorporated into clinical practice. The scores can serve multiple purposes for the clinicians. For example, they can be used for an initial admission risk assessment or continuous real-time risk monitoring.^52^ Each unit can establish thresholds that, when crossed, trigger alerts indicating early signs of deterioration, ensuring timely identification and rescue of patients at risk of adverse outcomes.^53^ Additionally, scores could inform triage decisions, such as whether to admit an ED patient to the ward or ICU or if an ICU patient can transition to a lower acuity setting. Finally, the health system can use unit-level pCREST scores to optimize the allocation of hospital resources.

The availability of a single pCREST risk score can significantly enhance communication among health care practitioners across units by establishing a shared mental model of patient risk, creating better clinician alignment regarding a patient’s health.^54^ Several studies have stressed the importance of effective communication and standardized protocols during patient handover.^50,55,56,57,58,59^ pCREST scores could play a pivotal role in these programs by providing objective measures of the severity of illness that can guide care coordination among teams.

Our variable importance analysis revealed that Fio_2_, heart rate, temperature, and respiratory rate were the most important in predicting pediatric critical illness.^60,61,62,63^ The hospital unit location of the patient was considered an additional influential variable. This further supports our hypothesis that our model uses the hospital unit as a proxy for adjustment according to patient acuity or complexity, allowing us to generalize across units. However, the reasons for ICU admission are likely to be multifactorial and may vary across hospital sites and time, indicating the importance of undertaking external validation, with and without temporal splits, before pCREST can be implemented in a hospital.

### Limitations

This study has several limitations. Our study is retrospective in nature and may have limitations in the data elements collected. For example, our model uses the number of prior comorbidities as a feature, which relies on the completeness and accuracy of recorded diagnosis codes for past visits in the EHR. Before pCREST implementation, future work should address ethical concerns with machine learning models, such as ensuring fairness of operation across relevant patient groups, data security, and implementing effective explainability algorithms at the bedside. Similar to our prior work,^48^ assessment of patient outcomes and evaluation of clinical utility after model implementation are also needed before wide adoption. Additionally, choosing critical events could fall short of a consensus outcome of deterioration throughout a pediatric hospital.^16^ Validation for other measures of hospital deterioration remains a focus of future work.

## Conclusions

In this retrospective cohort study, We developed and externally validated a new EHR-based machine learning model called pCREST for predicting critical events among hospitalized children across hospital EDs, wards, and ICUs. Our model could be used to monitor a child’s health seamlessly throughout their hospital journey, facilitating early recognition of deterioration and timely intervention.

## Notes

Supplement 1.
eMethods.
**eTable 1.** Distribution and Admission-Level Missingness Rates of All Evaluated Features for Each Patient Cohort **eTable 2.** A List of All Evaluated Hyperparameters, Search Values Considered, and the Optimal Hyperparameters Selected to Train Each Respective Model **eTable 3.** Internal and External Area Under the Precision-Recall Curve Metrics of All Models Evaluated in this Study **eTable 4.** Sensitivity and Specificity of Different Cutoffs for pCREST and pCART for Patients With a Critical Event Compared With Those Not Experiencing Any Event in the External Validation Data **eTable 5.** Comparison of the Performance of Gradient-Boosted Machine Models Trained Using Unit-Specific Data and pCREST for Predicting Pediatric Critical Events Within the Next 12 Hours in the External Test Data **eTable 6.** Performance of pCREST Within Individual Years From 2018-2021 Within the External Test Cohort **eTable 7.** Evaluation of pCREST Performance in Patient Subgroups Stratified by Patient Age Within the External Test Cohort **eTable 8.** Evaluation of pCREST Performance in Patient Subgroups Stratified by the Number of Prior Comorbidities Within the External Test Cohort **eFigure 1.** Illustration of the Discrete-Time Survival Analysis Framework for Blocking Derivation Data into 12-Hour Intervals for Deriving the Logistic Regression and the Gradient-Boosted Machine Learning Models **eFigure 2.** Explanation of pCREST Predictions Using Shapley Values for a Test Patient (Age 16 Years) From the UW-Madison Cohort **eFigure 3.** Efficiency Curves for Predicting Critical Events in the Next 12 hours Using pCREST in the External Validation Cohort for Each Unit

Supplement 2.
Data Sharing Statement
