# Supplementary material for: Machine Learning for Predicting Critical Events Among Hospitalized Children
Source: JAMA Netw Open. 2025 May 30;8(5):e2513149. doi: 10.1001/jamanetworkopen.2025.13149 (PMC12125637; doi:10.1001/jamanetworkopen.2025.13149)
Supplement: Supplement 1. — eMethods. eTable 1. Distribution and Admission-Level Missingness Rates of All Evaluated Features for Each Patient Cohort eTable 2. A List of All Evaluated Hyperparameters, Search Values Considered, and the Optimal Hyperparameters Selected to Train Each Respective Model eTable 3. Internal and External Area Under the Precision-Recall Curve Metrics of All Models Evaluated in this Study eTable 4. Sensitivity and Specificity of Different Cutoffs for pCREST and pCART for Patients With a Critical Event Compared With Those Not Experiencing Any Event in the External Validation Data eTable 5. Comparison of the Performance of Gradient-Boosted Machine Models Trained Using Unit-Specific Data and pCREST for Predicting Pediatric Critical Events Within the Next 12 Hours in the External Test Data eTable 6. Performance of pCREST Within Individual Years From 2018-2021 Within the External Test Cohort eTable 7. Evaluation of pCREST Performance in Patient Subgroups Stratified by Patient Age Within the External Test Cohort eTable 8. Evaluation of pCREST Performance in Patient Subgroups Stratified by the Number of Prior Comorbidities Within the External Test Cohort eFigure 1. Illustration of the Discrete-Time Survival Analysis Framework for Blocking Derivation Data into 12-Hour Intervals for Deriving the Logistic Regression and the Gradient-Boosted Machine Learning Models eFigure 2. Explanation of pCREST Predictions Using Shapley Values for a Test Patient (Age 16 Years) From the UW-Madison Cohort eFigure 3. Efficiency Curves for Predicting Critical Events in the Next 12 hours Using pCREST in the External Validation Cohort for Each Unit [file jamanetwopen-e2513149-s001.pdf]

Supplemental Online Content

Strutz S, Liang H, Carey K, et al. Machine learning for predicting critical events among hospitalized children. *JAMA Netw Open*. 2025;8(5):e2513149. doi:10.1001/jamanetworkopen.2025.13149

eMethods.
eTable 1. Distribution and Admission-Level Missingness Rates of All Evaluated Features for Each Patient Cohort
eTable 2. A List of All Evaluated Hyperparameters, Search Values Considered, and the Optimal Hyperparameters Selected to Train Each Respective Model
eTable 3. Internal and External Area Under the Precision-Recall Curve Metrics of All Models Evaluated in this Study
eTable 4. Sensitivity and Specificity of Different Cutoffs for pCREST and pCART for Patients With a Critical Event Compared With Those Not Experiencing Any Event in the External Validation Data
eTable 5. Comparison of the Performance of Gradient-Boosted Machine Models Trained Using Unit-Specific Data and pCREST for Predicting Pediatric Critical Events Within the Next 12 Hours in the External Test Data
eTable 6. Performance of pCREST Within Individual Years From 2018-2021 Within the External Test Cohort
eTable 7. Evaluation of pCREST Performance in Patient Subgroups Stratified by Patient Age Within the External Test Cohort
eTable 8. Evaluation of pCREST Performance in Patient Subgroups Stratified by the Number of Prior Comorbidities Within the External Test Cohort
eFigure 1. Illustration of the Discrete-Time Survival Analysis Framework for Blocking Derivation Data into 12-Hour Intervals for Deriving the Logistic Regression and the Gradient-Boosted Machine Learning Models
eFigure 2. Explanation of pCREST Predictions Using Shapley Values for a Test Patient (Age 16 Years) From the UW-Madison Cohort
eFigure 3. Efficiency Curves for Predicting Critical Events in the Next 12 hours Using pCREST in the External Validation Cohort for Each Unit

This supplemental material has been provided by the authors to give readers additional information about their work.

© 2025 Strutz S et al. *JAMA Network Open*.

eMethods

Data preparation for logistic regression (LR) and gradient-boosted (XGB) machine learning models: **eFigure 1** illustrates our approach. Briefly, observations in the derivation dataset were blocked into 12-hour time intervals, whereby the last recorded observation for each predictor was selected as the representative value per given time block. The models were then trained to predict the probability of the outcome within the next 12 hours of that observation.

Data preparation for deep learning models: The derivation data were unblocked for our deep learning models, keeping the variable-length time series natures of electronic health record (EHR) observations intact, similar to our prior work.[43] We included an "hours since admission" variable to enable the recurrent neural (RNN) models to be time-aware. The binary outcome for each observation was set to if the critical event occurred in the next 12 hours. All encounters within a single training batch were padded with a mask value to account for variable-length data, and a mask indicator was used to ensure models disregarded padded time steps.

Hyperparameter optimization: Hyperparameter optimization for the LR and XGB models was performed using a grid search and a five-fold cross-validation approach of the derivation dataset. We used Bayesian Optimization approaches to tune the hyperparameters of the RNN and XGB-RNN tandem models using an 80%-20% encounter-level split of the derivation dataset, similar to prior work.[43,44] The complete list of all evaluated hyperparameters and their optimal values for the non-deep learning and deep learning models are presented in **eTable 2**.

© 2025 Strutz S et al. *JAMA Network Open*.

Addressing missing values: Missing values for all models were addressed by first carrying forward the last recorded observation, followed by imputing unit-specific medians calculated from the derivation dataset for the LR and RNN models. No median imputation was done for the XGB models, as these could be trained with missing values.

Evaluation of pCREST against unit-specific models: We split our derivation data into ED, ward, and ICU-specific derivation datasets and created three unit-specific models. The hospital-wide model was then compared to each unit-specific model and to pCART for observations in the external test dataset filtered by ED, ward, or ICU units using AUCs.

[43] Bashiri FS, Carey KA, Martin J, et al. Development and external validation of deep learning clinical prediction models using variable-length time series data. J Am Med Inform Assoc. Published online April 29, 2024:ocae088. doi:10.1093/jamia/ocae088

[44] Mayampurath A, Bashiri F, Hagopian R, et al. Predicting neurological outcomes after in-hospital cardiac arrests for patients with Coronavirus Disease 2019. Resuscitation. 2022;178:55-62. doi:10.1016/j.resuscitation.2022.07.018

© 2025 Strutz S et al. *JAMA Network Open*.

eTable 1: Distribution and admission-level missingness rates of all evaluated features for each patient cohort.

Variable	UC Median (IQR)	UC % of patient admissions with missing values	Loyola Median (IQR)	Loyola % of patient admissions with missing values	UW-Madison Median (IQR)	UW-Madison % of patient admissions with missing values
Heart Rate	116 [94, 138]	0	116 [94, 138]	0	114 [92, 136]	0
Respiratory Rate	24 [20, 32]	0	24 [20, 32]	1	25 [20, 35]	0
Systolic Blood Pressure	103 [92, 115]	0	106 [97, 116]	1	106 [95, 117]	0
Diastolic Blood Pressure	60 [49, 71]	0	59 [52, 69]	1	59 [51, 68]	0
Oxygen Saturation	99 [97, 100]	0	98 [96, 99]	1	98 [96, 99]	0
Temperature	36.7 [36.3, 37.1]	0	36.9 [36.7, 37.4]	1	36.8 [36.6, 37.1]	0
Fraction of Inspired Oxygen	22 [21, 45]	1	21 [21, 29]	3	21 [21, 27]	0
AVPU	0 [0, 0]	45	0 [0, 0]	1	0 [0, 1]	10
Albumin	3.5 [3, 4]	62	3 [2.4, 3.6]	76	3 [2.5, 3.5]	71
Alkaline Phosphate	153 [100, 233]	62	126 [85, 187]	76	142 [98, 208]	72
Anion Gap	12 [10, 14]	38	9 [7, 11]	50	9 [7, 11]	41
SGOT	34 [23, 58]	62	33 [23, 50]	76	31 [20, 54]	70
Bands	5 [2, 11]	82	6 [2, 14]	89	30 [30, 30]	96
Bilirubin	0.3 [0.1, 0.6]	62	0.6 [0.4, 0.9]	76	0.4 [0.3, 0.9]	71
Blood Urea Nitrogen	10 [6, 16]	38	8 [5, 12]	50	10 [7, 16]	43
Calcium	9 [8.6, 9.5]	38	9 [8.5, 9.5]	51	9 [8.5, 9.5]	44
Creatinine	0.3 [0.2, 0.5]	38	0.42 [0.3, 0.6]	50	0.4 [0.3, 0.6]	41
Glucose	106 [92, 128]	37	106 [92, 125]	50	105 [91, 126]	42
Bicarbonate	24 [21, 27]	38	24 [21, 27]	50	24 [21, 27]	39
Hemoglobin	10.4 [9, 11.9]	39	10.8 [9.2, 12.3]	48	10.9 [9.4, 12.5]	42

© 2025 Strutz S et al. *JAMA Network Open*.

Lactate	1.1 [0.8, 1.6]	87	1.39 [1, 1.9]	94	1.1 [0.8, 1.6]	83
MCV	84.1 [79.7, 88.5]	39	84.7 [80.4, 88.8]	50	84 [80, 88]	44
Neutrophil	60 [44, 74]	58	64 [44, 78]	54	63 [46, 76]	51
Phosphate	4.2 [3.4, 5]	68	4.4 [3.6, 5.1]	83	4.3 [3.5, 5.1]	73
Platelet	255 [161, 355]	39	277 [197, 376]	50	248 [158, 352]	44
Potassium	4 [3.6, 4.4]	39	4 [3.6, 4.3]	49	4 [3.7, 4.3]	40
Total protein	6.1 [5.3, 6.9]	62	6 [5.2, 6.8]	76	6 [5.2, 6.9]	74
Sodium	140 [138, 143]	38	138 [136, 140]	49	139 [137, 141]	40
White Blood Cell Count	9.9 [6.7, 14.1]	39	9.7 [6, 14]	49	9.9 [6.7, 13.9]	44
Age	4 [1, 11]	0	5 [1, 13]	0	5 [0, 12]	0

IQR: Interquartile Range
AVPU: Alert Verbal Pain Unresponsive
SGOT: serum glutamic-oxaloacetic transaminase,
MCV: mean corpuscular volume

© 2025 Strutz S et al. *JAMA Network Open*.

eTable 2: A list of all evaluated hyperparameters, search values considered, and the optimal hyperparameters selected to train each respective model.

Model	Parameters	Search Values	Best
LR	Regularization parameter	0.0, 0.000005, 0.00001, 0.000011, 0.00002, 0.00004, 0.00005, 0.0001, 0.00015, 0.001, 0.005	0.0
	Mixing parameter	0.0, 0.3, 0.4, 0.45, 0.49, 0.5, 0.55, 0.7, 0.8, 1.0	1.0
XGB	Number Boosting Iterations	500, 1000, 1500	1000
	Maximum Tree Depth	2, 5, 10, 15	10
	Learning Rate/Shrinkage (eta)	0.001, 0.01, 0.1	0.01,
	Minimum Sum of Instance Weight	1, 2	2
	Subsample Percentage	0.5	0.5
RNN & (XGB-RNN)	Recurrent cell type	LSTM, GRU	GRU (LSTM)
	Recurrent cell units	[20,100]	50 (60)
	Number of additional dense layers	1, 2	2 (1)
	Dense cell units	[20, 80]	30 (48)
	Dropout	[0.0, 0.25]	0.046 (0.0)
	Optimizer	Adam	Adam (Adam)
	Learning rate	[0.0005, 0.005]	0.0015 (0.005)
	Include learning rate decay	True, False	True (True)
	Learning rate decay rate	[0.7, 0.99]	0.92 (0.7)
	Learning rate decay number of steps	[300,500]	309 (500)
	Batch size	16, 32, 64	32 (16)
	Time quantile threshold	0.95, 0.97, 0.99	0.99 (0.99)

LR: Logistic Regression; XGB: Gradient Boosted Machine; RNN: Recurrent Neural Networks
LSTM: Long-Short Term Memory Unit; GRU: Gated Recurrent Unit

© 2025 Strutz S et al. *JAMA Network Open*.

eTable 3: Internal and external Area Under the Precision-Recall Curve (AUPRC) metrics (with 95% CI) of all models evaluated in this study.

Model	AUPRC (95% CI)	
	Temporal Validation	External Validation
Bedside PEWS	0.07 (0.07, 0.08)	0.04 (0.04, 0.04)
pCART	0.08 (0.08, 0.08)	0.07 (0.07, 0.07)
Logistic Regression	0.12 (0.11, 0.12)	0.11 (0.11, 0.11)
XGBoost (pCREST)	0.19 (0.18, 0.19)	0.16 (0.15, 0.16)
RNN	0.14 (0.14, 0.15)	0.12 (0.11, 0.12)
XGBoost-RNN	0.17 (0.16, 0.17)	0.11 (0.11, 0.12)

© 2025 Strutz S et al. *JAMA Network Open*.

eTable 4: Sensitivity and specificity of different cut-offs for pCREST and pCART for patients suffering a critical event compared with those not experiencing any event in the external validation data. Cut-offs for both models are represented as predicted probabilities x 1000. At a sensitivity of 73%, pCREST had a higher specificity than pCART (82% vs. 75%). At a specificity of 85%, pCREST demonstrated a higher sensitivity than pCART (69% vs. 59%).

Model Cutoff	Sensitivity (%, 95%CI)	Specificity (%, 95%CI)
**pCREST (prob X1000)**		
≥ 0.7	97 (97-98)	34 (34-34)
≥ 1.0	95 (95-95)	46 (46-46)
≥ 1.2	94 (94-94)	50 (50-50)
≥ 1.9	91 (91-92)	60 (60-60)
≥ 3.3	85 (85-86)	71 (71-71)
≥ 4.1	82 (82-82)	75 (75-75)
≥ 5.1	78 (78-79)	78 (78-78)
≥ 6.8	73 (73-74)	82 (82-82)
≥ 8.7	69 (69-70)	85 (85-85)
≥ 11.2	64 (63-64)	88 (88-88)
≥ 14.0	59 (59-60)	90 (90-90)
≥ 18.5	53 (53-54)	92 (92-93)
≥ 27.1	45 (45-46)	95 (95-95)
≥ 42.8	36 (35-37)	97 (97-97)
**pCART (prob X1000)**		
≥ 2.0	97 (97-97)	27 (27-27)
≥ 2.5	95 (95-95)	37 (36-37)
≥ 3.2	92 (92-92)	46 (46-46)
≥ 3.7	90 (90-90)	51 (51-51)
≥ 5.1	85 (85-85)	61 (61-61)
≥ 7.3	78 (78-79)	71 (71-71)
≥ 8.7	73 (73-74)	75 (75-75)
≥ 10.6	68 (68-69)	79 (79-79)
≥ 12.5	64 (63-64)	82 (82-82)
≥ 15.2	59 (59-60)	85 (85-85)
≥ 19.4	53 (53-54)	88 (88-88)
≥ 24.2	48 (47-49)	90 (90-90)
≥ 47.5	34 (34-35)	95 (95-95)
≥ 76.9	24 (24-25)	97 (97-97)

© 2025 Strutz S et al. *JAMA Network Open*.

eTable 5: Comparison of the performance of gradient-boosted machine (XGB) models trained using unit-specific data and pCREST for predicting pediatric critical events within the next 12 hours in the external test data. In addition, pCREST performance was superior or comparable to pCART in predicting critical events depending on the unit (AUC in the ED: 0.80 vs. 0.69, P<0.001; ward: 0.78 vs. 0.79, P<0.001; and ICU: 0.75 vs. 0.71, P<0.001).

Location	Unit-Specific Model AUC (95% CI)	pCREST AUC (95% CI)
ED-only	0.80 (0.79, 0.80)	0.80 (0.79, 0.80)
WARD-only	0.76 (0.76, 0.77)	0.78 (0.77, 0.79)*
ICU-only	0.74 (0.74, 0.75)	0.75 (0.75, 0.76)*

*P<0.001 compared with the unit-specific models

© 2025 Strutz S et al. *JAMA Network Open*.

eTable 6: Performance of pCREST within individual years from 2018-2021 within the external test cohort. No observable differences in performance were noted before (2018, 2019) and during (2020, 2021) the COVID-19 pandemic years.

External Test Dataset Year	Number of patient admissions	Number of patient admissions with critical events (%)	pCREST AUC (95% CI)
2018	3,593	196 (5.4%)	0.87 (0.86, 0.87)
2019	3,307	194 (5.9%)	0.85 (0.85, 0.86)
2020	2,713	209 (7.7%)	0.85 (0.84, 0.86)
2021	2,968	192 (6.5%)	0.86 (0.85, 0.86)

eTable 7: Evaluation of pCREST performance in patient subgroups stratified by patient age within the external test cohort. Age categories were based on our prior work (Mayampurath et al., Pediatric Critical Care Medicine 2020, PMCID: PMC7483876). pCREST performance was equivalent across patients in all age groups (AUC range 0.86-0.87), except in children younger than 2 years (AUC 0.83).

Age	Number of patient admissions	Number of patient admissions with critical events (%)	pCREST AUC (95% CI)
Infant [< 2 years]	11,300	1120 (9.9%)	0.83 (0.83, 0.83)
Preschool [2-5 years]	8,765	457 (5.2%)	0.86 (0.85, 0.86)
School-Age [6 -11 years]	9,184	444 (4.8%)	0.88 (0.88, 0.88)
Adolescent [> 11 years]	12,400	522 (4.2%)	0.87 (0.86, 0.87)

eTable 8: Evaluation of pCREST performance in patient subgroups stratified by the number of prior comorbidities within the external test cohort. Performance was equivalent (AUC range 0.85-0.87) across subgroups stratified by the number of prior comorbidities.

Number of Prior Comorbidities	Number of patient admissions	Number of patient admissions with critical events (%)	pCREST AUC (95% CI)
0	20,850	707 (3.39)	0.87 (0.87, 0.88)
1	6,267	465 (7.42)	0.87 (0.86, 0.87)
>1	14,532	1371 (9.43)	0.85 (0.85, 0.85)

© 2025 Strutz S et al. *JAMA Network Open*.

eFigure 1: Illustration of the discrete-time survival analysis framework for blocking derivation data into 12-hour intervals for deriving the logistic regression and the gradient-boosted machine learning models. Before blocking, the EHR observations (vital signs, laboratory results) are recorded at irregular intervals. During blocking, the most recent observations are carried forward to the end of the block. The outcome for that time block is set to 1 if the outcome occurs in the next block and 0 otherwise. The time-blocked observations are then used to train the model. Note that the deep learning models were trained on non-blocked irregular sampled data.

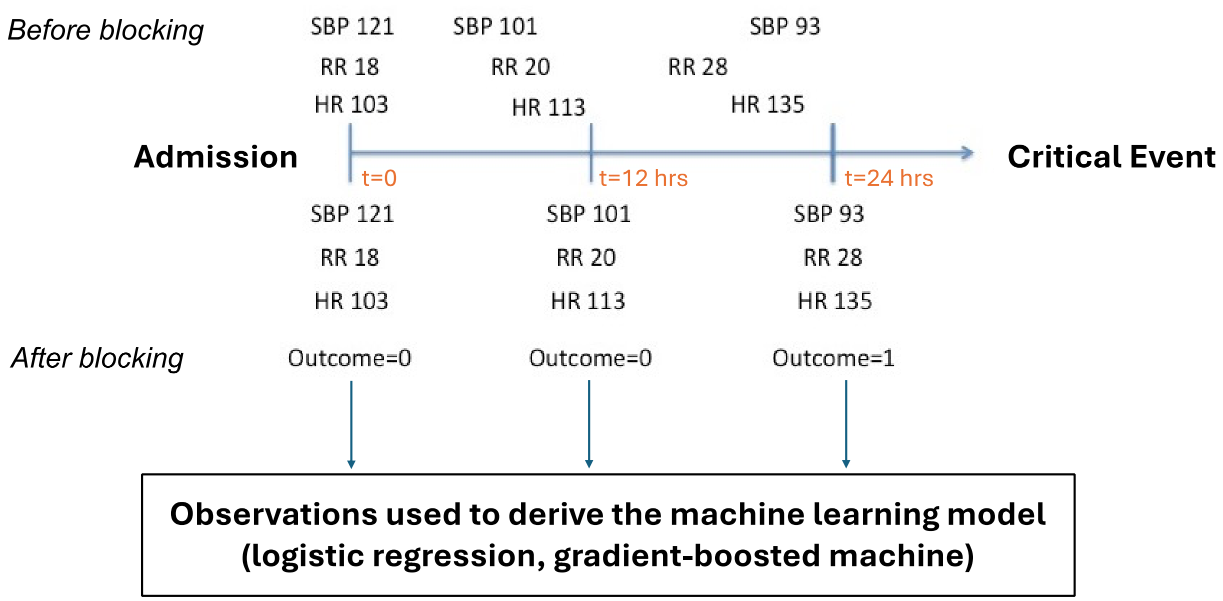

© 2025 Strutz S et al. *JAMA Network Open*.

eFigure 2: Explanation of pCREST predictions using Shapley values for a test patient (age 16 years) from the UW-Madison cohort. During the ED, at approximately four hours after arrival, the age, lack of AVPU, and having more than one prior comorbidity were important to pCREST scoring in the 85th percentile. The patient appears to have stabilized upon being transferred to the ward. However, at approximately hour 88 of hospital stay, the pCREST percentile soared to 100, and Shapley values indicate the fraction of inspired oxygen (FiO2), aspartate aminotransferase test (AST), and oxygen saturation values as being important to the high pCREST score. The increased risk persisted upon transfer to the intensive care unit at hour 98 (approximately 20 hours before experiencing a critical event), with FiO2 and location being important to the pCREST percentile score of 94.

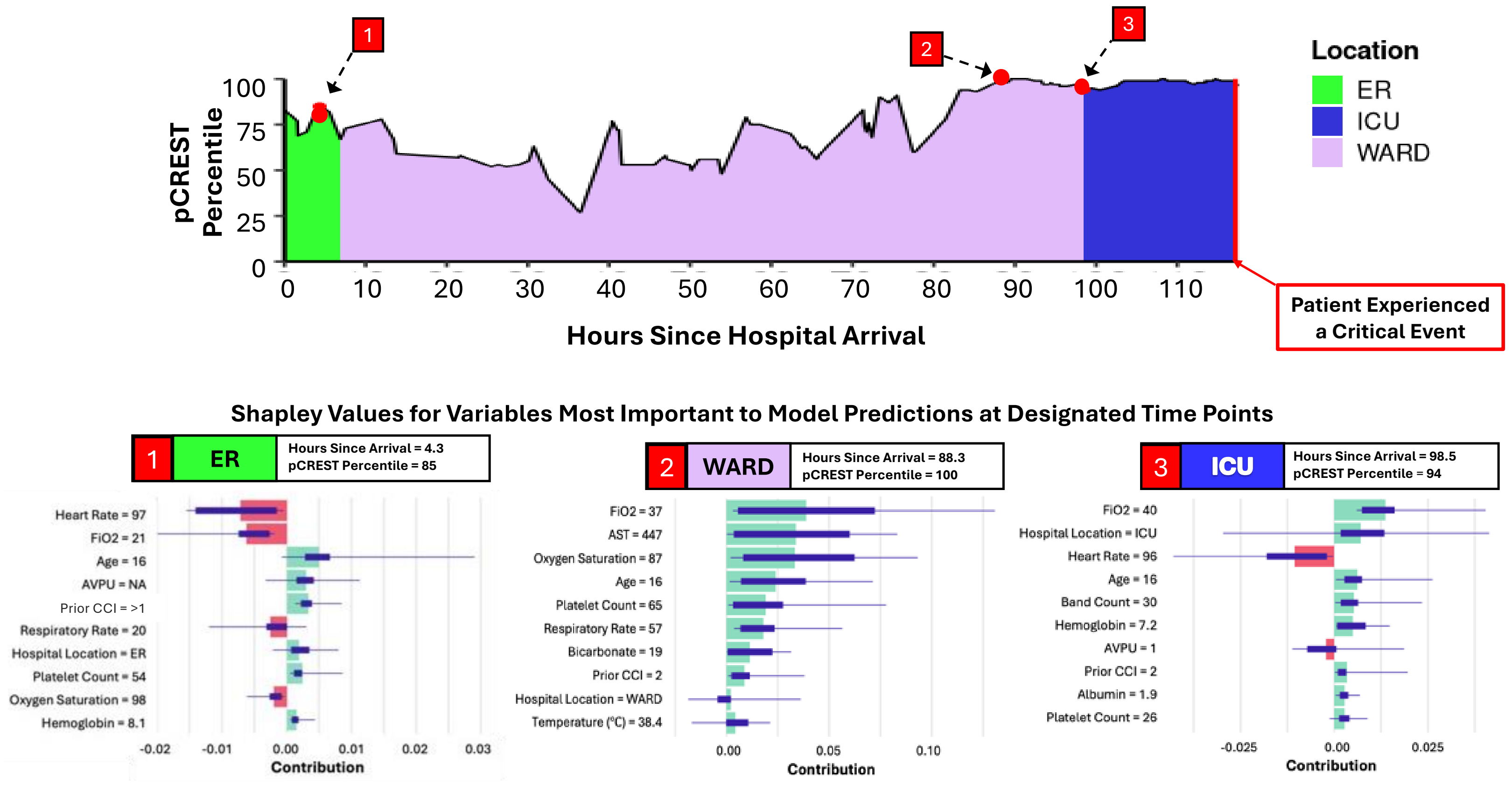

© 2025 Strutz S et al. *JAMA Network Open*.

eFigure 3: Efficiency curves for predicting critical events in the next 12 hours using pCREST in the external validation cohort for each unit. Each line indicates the percentage of observations with positive alerts against pCREST score sensitivity for different score values for that unit.

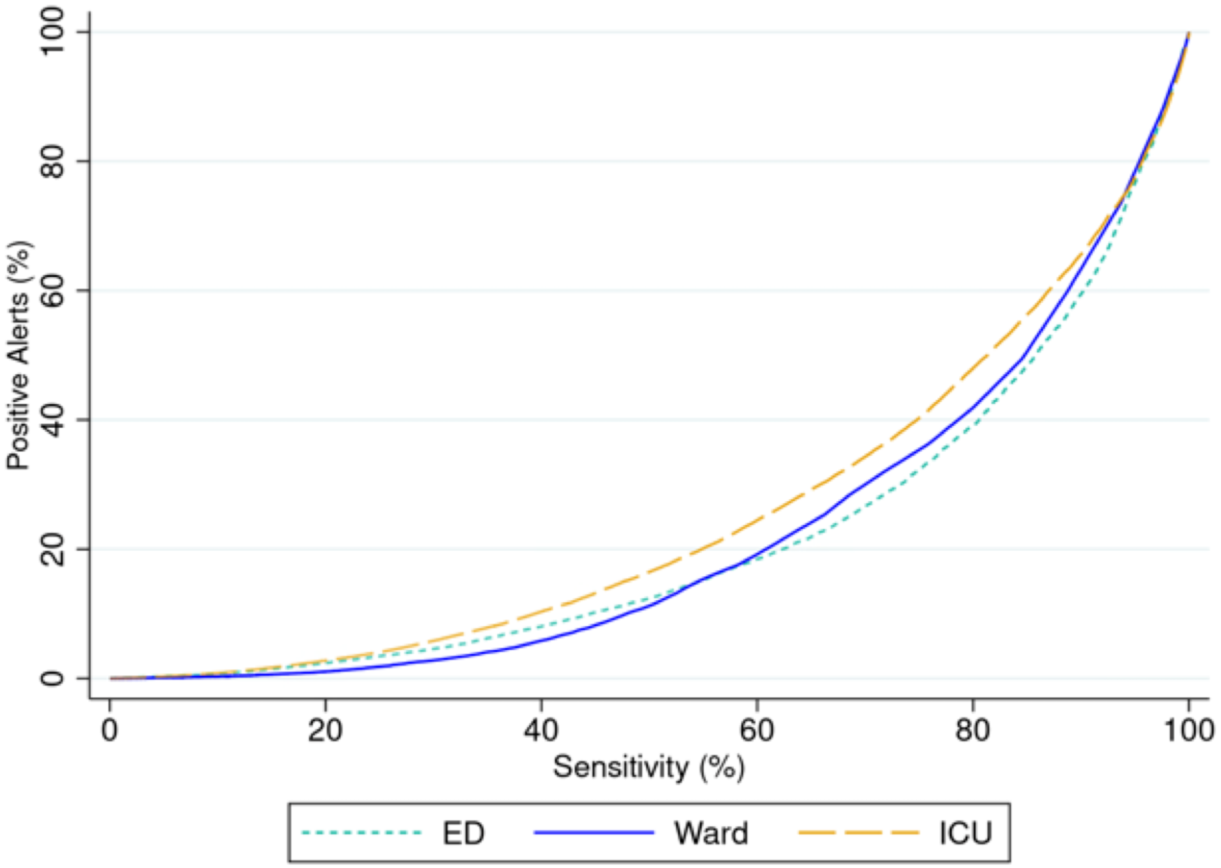

© 2025 Strutz S et al. *JAMA Network Open*.
