# Supplementary material for: Machine Learning for Predicting Critical Events Among Hospitalized Children
Source: JAMA Netw Open. 2025 May 30;8(5):e2513149. doi: 10.1001/jamanetworkopen.2025.13149 (PMC12125637; doi:10.1001/jamanetworkopen.2025.13149)
Supplement: Supplement 2. — Data Sharing Statement [file jamanetwopen-e2513149-s002.pdf]

Data Sharing Statement

Strutz. Machine Learning for Predicting Critical Events Among Hospitalized Children. *JAMA Netw Open*. Published May 30, 2025. doi:10.1001/jamanetworkopen.2025.13149

Data
Data available: No
